# Characteristics Analysis, Clinical Outcome and Risk Factors for Fungal Peritonitis in Peritoneal Dialysis Patients: A 10-Year Case-Control Study

**DOI:** 10.3389/fmed.2021.774946

**Published:** 2021-12-01

**Authors:** Rongrong Li, Difei Zhang, Jingwen He, Jianjun Ou, La Zhang, Xiaoxuan Hu, Jianfeng Wu, Hui Liu, Yu Peng, Yuan Xu, Haijing Hou, Xusheng Liu, Fuhua Lu

**Affiliations:** ^1^The Second Clinical College of Guangzhou University of Chinese Medicine, Guangzhou, China; ^2^Department of Nephrology, Guangdong Provincial Hospital of Chinese Medicine, Guangzhou, China

**Keywords:** fungal peritonitis, antifungal susceptibility, antifungal treatment, clinical outcome, risk factor, peritoneal dialysis

## Abstract

**Background:** Fungal peritonitis (FP) is a rare but severe complication that can appear in patients receiving peritoneal dialysis (PD). This study aimed to investigate the incidence rate and clinical characteristics of FP, evaluate clinical outcomes between FP and bacterial peritonitis (BP) patients on PD, and especially estimate the risk factors for FP outbreak.

**Methods:** All episodes of FP diagnosed in our hospital from January 1, 2011, to December 31, 2020, were reviewed in this single-center study. FP cases were analyzed and compared with patients diagnosed with BP in a 1:6 ratio matching for case-control study. Patient information, including clinical information, biochemical analysis, and outcomes, was recorded. Univariate and multivariate logistic regression model were used to analyze the risk factors for FP.

**Results:** A total of 15 FP episodes were observed in 15 PD patients, with an FP rate of 0.0071 episodes per patient-year. Seventeen strains of fungi were isolated and identified. *Candida* was the most common pathogen (15 strains, 88.2%), followed by *Aspergillus fumigatus* (2 strains, 11.8%). Between the groups, FP group showed a higher rate of HD transfer and catheter removal, and a lower rate of PD resumption in the short-term outcome (all *P* < 0.01), while no significant difference in the mortality was noted during the whole study period. The multivariate logistic regression analysis showed that longer PD duration (odds ratio [OR] 1.042, 95% confidence interval [CI] 1.012–1.073, *P* < 0.01), higher serum potassium (OR 3.373, 95% CI 1.068–10.649, *P* < 0.05), elevated estimated glomerular filtration rate (eGFR) (OR 1.845, 95% CI 1.151–2.955, *P* < 0.05), reduced serum albumin level (OR 0.820, 95% CI 0.695–0.968, *P* < 0.05) and peritoneal effluent polymorphonuclear (PMN) count (OR 0.940, 95%CI 0.900–0.981, *P* < 0.01) were significantly increased the risk for FP.

**Conclusion:** These results suggested that FP leads to higher rate of catheter removal and HD transfer, and a lower rate of PD resumption than BP, and that additional attention should be paid to hypoalbuminemia, increased serum potassium, long PD duration, and low peritoneal effluent PMN in PD patients.

## Introduction

Peritoneal dialysis (PD) is a widely accepted renal replacement therapy for end-stage renal disease (ESRD) patients (1, 2). In recent years, with the development of the national economy and the continuous improvement of medical insurance policy, the number of patients on PD has increased dramatically (3). However, PD-associated peritonitis (PDAP), one of the most common and severe complications of PD, is the leading cause of technical failure and hospitalization, causing deaths in 5–16% of PD patients (4, 5). Peritonitis can be caused mainly by bacteria (bacterial peritonitis; BP) and fungi (fungal peritonitis; FP). FP is relatively rare compared to BP, accounting for only 1–12% of patients with PDAP (6–12). It was reported that antibiotic use history, prolonged PD duration, malnutrition or hypoalbuminemia, and diabetes are usually identified as important risk factors for the occurrence of FP (6, 13). FP is related to high hospitalization rates, catheter removal, permanent transfer to HD, and death (12, 14), and the treatment method is relatively limited.

Current guidelines and literature (5, 15, 16) indicate that immediate catheter removal combined with antifungal therapy after FP diagnosis results in the best overall outcome and is considered the best strategy to improve survival in patients with FP. Unfortunately, early diagnosis of FP remains challenging due to the lack of specificity in the clinical manifestations of FP, the technical delay in microbiological diagnosis (15, 16), and the differences in socioeconomic environment and clinical management (7, 9). Although many retrospective studies on FP have been reported worldwide, the disease characteristics, treatment, and prevention methods of FP are still controversial, the potential risk factors of FP occurrence remain unclear, and little attention has been paid to the outcome differences of FP and BP.

This study retrospectively analyzed all FP cases in our PD center and matched FP in a 1:6 ratio with BP cases. Our primary objective was to investigate the incidence rate of FP, microbiological testing, clinical features, and antifungal therapy. Our secondary objective was to compare the clinical outcomes and identify risk factors that may discriminate between FP from BP in patients on PD.

## Patients and Methods

### Study Design and Objectives

This is a single-center, retrospective, observational, case-control study. Between January 1, 2011, and December 31, 2020, all patients receiving PD treatment at the PD center of Guangdong Provincial Hospital of Chinese Medicine, the Second Affiliated Hospital of Guangzhou University of Chinese Medicine, Guangzhou, China, were retrospectively evaluated. Inclusion criteria were patients with ESRD who received PD therapy, were over 18 years of age at the time of PD, and were followed at our PD center. Patients who were unwilling to participate or could not be followed up at our center, patients who had incomplete baseline data and previously received kidney transplant, or those who transferred from HD for more than 3 months were excluded. All enrolled patients were followed up either until December 31, 2020, cessation of PD or death.

Referring to the recommendations of the International Society for Peritoneal Dialysis (ISPD) in 2016 (5), peritonitis is diagnosed if: (1) clinical features are consistent with peritonitis, namely abdominal pain and/or cloudy dialysis effluent; (2) dialysis effluent white cell count (EWCC)>100/μl, with > 50% polymorphonuclear (PMN); and (3) dialysis effluent culture is positive. PD-associated peritonitis was defined as fungal peritonitis if positive for at least one fungal culture. Episodes per patient-year were used to calculate and report the incidence rate of any type of peritonitis.

**Figure 1:**
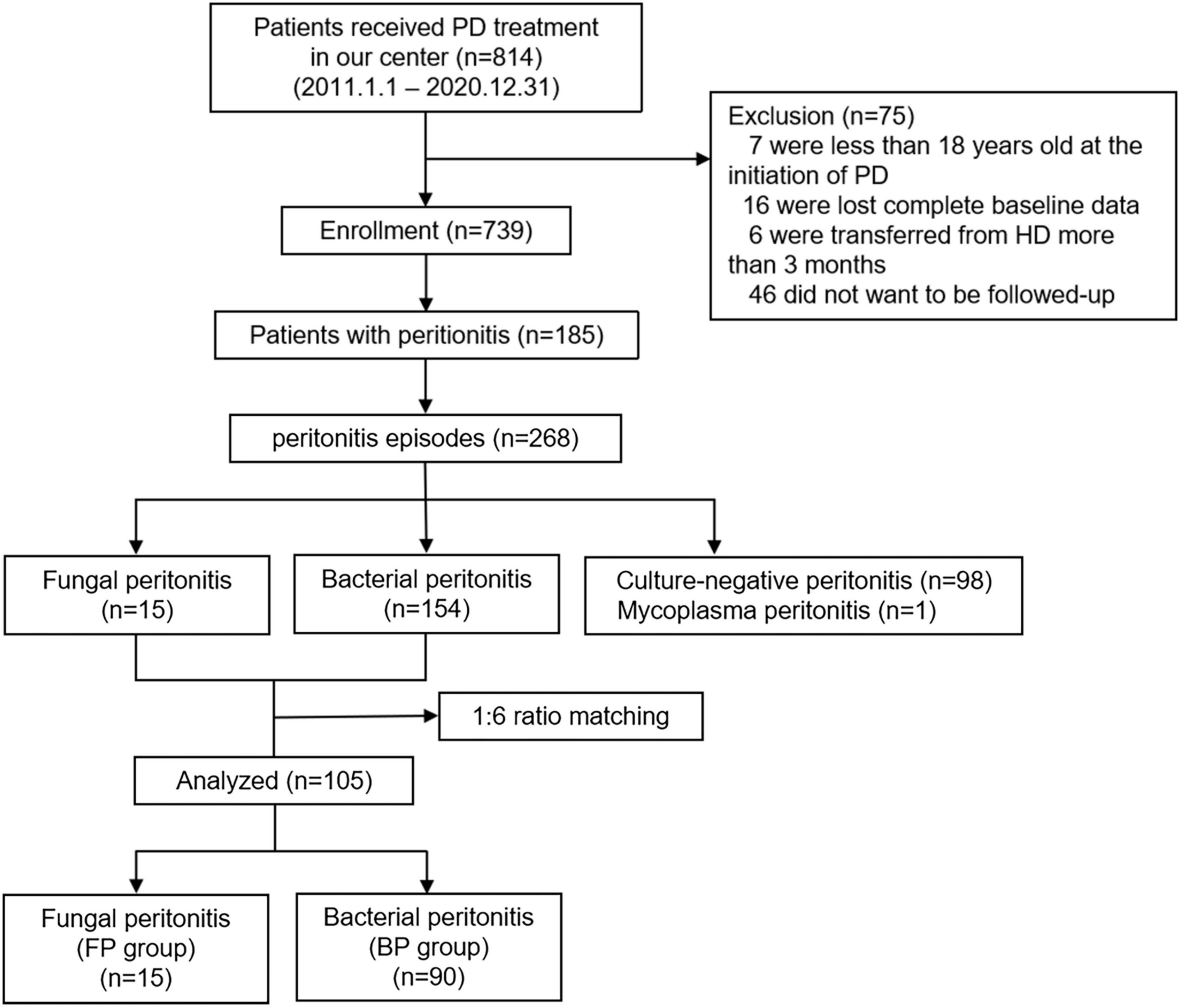
Study flow diagram.

To identify unique risk factors of FP, all FP cases were matched in a 1:6 ratio with those of BP. Finally, 105 cases of peritonitis were included in the study: 15 patients in the FP group and 90 patients in the BP group. The study design is depicted in Figure 1.

All the procedures of this study have been approved by the Institutional Ethics Review Boards of Guangdong Provincial Hospital of Chinese Medicine, The Second Affiliated Hospital of Guangzhou University of Chinese Medicine, Guangzhou, China (ZE2019-155-01), as well as the Declaration of Helsinki of 1964 and subsequent amendments or comparable ethical standards. All patients were asked for permission to use their medical data for non-commercial study, and provided their written informed consent at the initiation of PD therapy.

### Data Collection

The Data were retrospectively obtained from the electronic medical registration system (outpatient and inpatient medical records and laboratory database) of Guangdong Provincial Hospital of Chinese Medicine (China) and the PD center registry.

Among all peritonitis cases, the following clinical data were recorded: sex, age, cause of ESRD, body mass index (BMI), PD duration (the time from the start of PD to the onset of peritonitis), 24 h urine output, laboratory examination [peritoneal effluent white cell count (WCC), peritoneal effluent PMN, potassium, calcium, phosphorus, glucose, serum creatine, blood urea nitrogen, serum albumin (ALB), hemoglobin (Hb), intact parathyroid hormone (iPTH), blood WCC, high sensitivity C-reactive protein (hsCRP)] on admission and clinical outcome (death, HD transfer, PD resumption, kidney transplant, and loss of follow-up). PDAP-related death was defined as death due to active PDAP, or within 4 weeks of the occurrence of PDAP, or during hospitalization for PDAP. Clinical outcome within 3 months after diagnosis of FP was classified as short-term outcomes, and long-term outcomes refers to the clinical outcome after 3 months until the end of follow-up. We collected the exact causes of death, PD resumption, HD transfer, and catheter removal.

All fungal peritonitis episodes were also reviewed for clinical symptoms (cloudy effluent, abdominal pain, fever, gastrointestinal symptoms such as vomiting, diarrhea, nausea, or abnormalities in stool examination), potential risk factors for FP reported in other literatures (immunosuppressive therapy, presence of diabetes, previous antibiotic treatment, previous episodes of peritonitis, or other non-intraperitoneal infections), organism identification and antifungal susceptibility of FP, antifungal treatment, PD modality, and hospitalization. Non-intraperitoneal infections refer to the extra infections found after admission due to FP, except for the peritoneal infections, such as bowel-source infection and gynecological-source infection. Previous antibiotic treatment was defined as use of antibiotic treatment, whether orally, intravenously or intraperitoneally, for peritonitis or for any other infection in a month prior to the FP.

### Microbiological Investigations

After admission to the hospital, patients suspected of PDAP were treated with aseptic procedures for peritoneal effluent fluid retention before antibiotic treatment was initiated. First, 10 ml of effluent fluid was kept in the urine culture cup for routine effluent fluid examination and smear for pathogenic bacteria examination. At the same time, 30 ml of effluent fluid was retained in the blood culture flask (aerobic and anaerobic) for bacterial and fungal culture. Second, antifungal susceptibility tests were carried out in patients with a positive fungal culture.

Isolate identification, antifungal susceptibility tests, and other microbiological procedures were performed independently by the Guangdong Provincial Hospital of Traditional Chinese Medicine Laboratory Department. All yeast from PD effluents were isolated on Sabouraud's Dextrose Agar (SDA) and incubated at both 25 and 37°C for 7days aerobically. Matrix Assisted Laser Desorption Ionization Time-of-Flight VITEK MS was used to perform the identification of the yeasts to the species level automatically, which offer rapid and accurate recognition of a broad range of pathogenic yeasts. The antifungal susceptibility to amphotericin B, 5-flucytosine, fluconazole, voriconazole, and itraconazole of each strain was fully automated using the ATB FUNGUS 3 susceptibility card (bioMerieux). The analysis and interpretation of results were performed according to the criteria of the M27-A2 guidelines of the National committee for clinical laboratory (NCCLS) (17).

### Management Protocol of BP

Timely removal of catheters, empirical selection of antifungal drugs, targeted antifungal programs and adjuvant treatment are the main treatment measures of our center, based on the patient's clinical status and etiological results, according to ISPD guidelines and combined with our center's experience.

### Statistical Analysis

Normally distributed continuous variables, skewed continuous variables, and categorical variables were respectively expressed as Mean ± SD ($\bar{x}$ ± s), Median (P25, P75), or frequencies (percentages). Student's t-test for parametric data, Mann-Whitney U test for non-parametric data, and Chi-square test for categorical data between groups were used as appropriate to evaluate the differences between FP and BP groups.

For comparison's sake, all 15 FP episodes were matched in a 1:6 ratio with 90 PD episodes diagnosed with BP, which occurred closely to each FP episode in time. The data for 1:6 matching was obtained from electronic medical system in hospital and merged to the registration database in our PD center. The 1:6 ratio matched cohort was used to analyze the clinical outcomes and risk factors between FP and BP.

Factors that influenced FP were screened by univariate and multivariate logistic regression analyses. First, univariate logistic regression analysis was performed. The variables included in the univariate logistics regression model were age, sex, BMI, PD duration, diabetes, cardiovascular disease (CVD) history, under immunosuppressive therapy, previous BP, previous antibiotic use, serum potassium, hemoglobin, serum albumin, blood uric acid, eGFR, iPTH, hsCRP, peritoneal effluent PMN and season change. Because the lowest incidence of peritonitis occurred during winter, the winter group was treated as the reference group. Covariates with *P* < 0.3 in the univariate logistics analysis, and demographic variables (for example age, sex and BMI), as well as PD duration, diabetes, CVD history, serum potassium and hemoglobin (because these are the important factors affecting occurrence of PD patients with peritonitis), were used for multivariate logistics regression. Results were expressed as the odds ratio (OR) and 95% confidence intervals (CI).

Prism8 (GraphPad Software Inc., La Jolla, CA) and IBM SPSS^®^ Statistics (v26) were used for statistical analysis. All tests were performed two-tailed, and *P* < 0.05 was considered statistically significant.

## Results

### The Overall Peritonitis Rate and FP Incidence Rate

During the follow-up period, a total of 814 patients received PD at our center. Seven of these patients were underaged at the time of PD. Six incident patients received HD for more than 3 months. Follow-up was refused in 46 patients after catheter implantation. Sixteen patients were excluded due to the loss of baseline data. Finally, we enrolled 739 patients in our study. We recorded 268 episodes of peritonitis in 185 patients on PD, with a cumulative follow-up of 25,260 patient-months, showing a peritonitis rate of 0.127 episodes per patient-year. Among all episodes of peritonitis, 15 episodes (5.6%) of FP, 154 episodes (57.5%) of BP, 1 episode (0.4%) of mycoplasma and 98 episodes (36.5%) of culture-negative peritonitis had been identified. The FP rate was calculated as 0.0071 episodes per patient-year, and it decreased from 0.0170 episodes per patient-year to 0.0051 episodes per patient-year between the first (2011–2015) and the second (2016–2020) 5-year periods.

### Demographic and Clinical Characteristics of FP

These 15 FP episodes occurred in 15 PD patients. A total of 4 episodes of FP had a previous history of BP, and these FP patients experienced 1.75 ± 0.5 (range 1–2) BP episodes before FP, most frequently due to *Klebsiella pneumoniae* and to a lesser extent due to *Acinetobacter baumannii*.

On admission, cloudy effluent, abdominal pain, and fever were present in 100, 93.3, and 66.7% of the patients, respectively. Non-intraperitoneal infection was found in six patients (40%), including pulmonary infection (4/6), septicemia (1/6), and cholecystitis (1/6). All FP cases had a history of antibiotic use in the past 1 month, of which 11 were due to pure peritonitis, 3 to pulmonary infection followed by peritonitis, and 1 to ruptured catheters followed by peritonitis. Detailed information on these 15 FP episodes is listed in Table 1 and Supplementary Table S1.

**Table 1:** Detailed information on 15 cases of fungal peritonitis.

**Variables**	***n* (%) or Median (P25, P75)**
**Primary cause for ESRD**	
CGN	9 (60.0)
DKD	3 (30.0)
IgAN	1 (6.7)
FSGS	1 (6.7)
BAN	1 (6.7)
**Clinical symptoms**	
Cloudy effluent	15 (100.0)
Abdominal pain	14 (93.3)
Fever	10 (66.7)
Gastrointestinal symptom	8 (53.3)
**Laboratory examination**	
Peritoneal effluent WCC (10^6^/L), median (IQR)	1,020 (520, 2,100)
Peritoneal effluent PMN (%), median (IQR)	80 (65, 88)
Hypotension	2 (13.3)
Hypoalbuminemia	8 (53.3)
Anemia	8 (53.3)
Hypokalemia	4 (26.7)
**Peritonitis risk factors**	
Previous bacterial peritonitis	4 (26.7)
Previous antibiotic treatment	15 (100.0)
Under immunosuppressive therapy	2 (13.3)
Non-intraperitoneal infections	6 (40.0)
**Treatment**	
Fluconazole	14 (93.3)
Levofloxacin	1 (6.7)
**CAPD**	15 (100.0)
**Hospitalization (days)**, median (IQR)	18 (10, 38)
**Median duration until PD catheter**	3 (0, 6)
**removal (days)**, median (IQR)	

*CGN, chronic glomerulonephritis; DKD, diabetic kidney disease; IgAN, immunoglobulin a nephropathy; FSGS, focal segmental glomerulosclerosis; BAN, benign arteriolar nephrosclerosis; IQR, interquartile range; FP, fungal peritonitis; CAPD, continuous ambulatory peritoneal dialysis; PD, peritoneal dialysis*.

### Causative Organisms for FP

Among the 15 FP episodes, 17 strains of fungi were identified, including 9 cases of single fungal infection and 6 cases of mixed infection. In the mixed infection group, there were 4 cases of fungal infection combined with bacterial infection and two cases with a mix of two types of fungal infection.

As shown in Table 2, *Candida* species were the most commonly represented fungus with 15 cases (88.2%): *C. albicans* (7/15), *C. parapsilosis* (4/15), *Torulopsis glabrata* (2/15), *C. pulcherrima* (1/15), and *C. famata* (1/15). In addition, two cases (11.8%) of *Aspergillus fumigatus* were reported. The results of the organism identification and antifungal susceptibility tests are shown in Table 2.

**Table 2:** Results of susceptibility of isolates from 15 cases of fungal peritonitis.

Organism identification (*n*)^*^	**Antifungal susceptibility testing (** ***n*** **)**														
	**Amphotericin B**			**Fluconazole**			**5-Flucytosine**			**Voriconazole**			**Itraconazole**		
	**S**	**R**	**I**	**S**	**R**	**I**	**S**	**R**	**I**	**S**	**R**	**I**	**S**	**R**	**I**
*Candida albican (7)*	7	0	0	7	0	0	6	0	1	5	0	2	7	0	0
*Candida parapsilosis (4)*	4	0	0	4	0	0	4	0	0	4	0	0	4	0	0
*Torulopsis glabrata (2)*	2	0	0	2	0	0	2	0	0	2	0	0	2	0	0
*Aspergillus fumigatus (2)*	ND			ND			ND			ND			ND		
*Candida pulcherrima (1)*	1	0	0	1	0	0	1	0	0	1	0	0	1	0	0
*Candida famata (1)*	1	0	0	1	0	0	1	0	0	1	0	0	1	0	0

*S, susceptible; R, resistant; I, intermediate; ND, not detected*.

* *Two types of fungi were detected in two patients*.

The antifungal susceptibility of the other 15 strains of fungi, except for two strains of *Aspergillus fumigatus*, could be determined. One isolated case of *C. albicans* was intermediate to 5-flucytosine, itraconazole, and susceptible to fluconazole, voriconazole, and amphotericin B. Another case of *C. albicans* was intermediate to itraconazole, while susceptible to fluconazole, voriconazole, 5-flucytosine, and amphotericin B. The other 13 strains were all susceptible to fluconazole, voriconazole, 5-flucytosine, itraconazole, and amphotericin B.

### Treatment of FP

None of the patients at our center had received antifungal prophylaxis. PD was stopped immediately after the definitive diagnosis of FP. Fourteen patients were treated with fluconazole for at least 14 days at a dose of 200 mg every 24 h, after a loading dose of 400 mg. Among those, IV fluconazole was administrated to five patients, four patients initially received oral fluconazole, four patients received IV and IP fluconazole, and a combination of oral and IP fluconazole was administrated to one patient. This patient, treated with levofloxacin (500 mg/24 h) for 21 days, was infected with Aspergillus fumigatus, which did not have a sensitivity test. Six patients were treated with multiple antibiotics in addition to antifungal therapy because of concomitant bacterial infections. The median hospital stay for all FP was 18 days [10, 38].

### Comparison of Clinical Outcomes Between FP and BP in the 1:6 Ratio Cohort

To further analyze the outcomes and identify the risk factors of FP occurrence, 15 cases of FP were matched in a 1:6 ratio with those of BP (Table 3; Supplementary Table S2). Regarding the short-term clinical outcomes within 3 months of FP diagnosis, 13 FP cases (86.7%) undergone PD catheter removal after a median of 3 days [0, 6] of delay, and the exception two cases (13.3%) refused to remove the catheter. Only ten cases (11.1%) removed PD catheters in the BP group. The rate of catheter removal was much higher in FP group than that in BP group (*P* < 0.01) between the two groups. Ten cases in the FP group (66.7%) and eight cases in the BP group (8.9%), who survived after peritonitis episode, shifted to HD more than 3 months. A total of 84 cases continued on PD with an uneventful recovery, of which three cases (20%) were from the FP group, and 81 cases (93%) were from the BP group. Only two patients died within 3 months after FP and BP diagnosis, respectively (Table 4). It is noteworthy that FP was not the immediate cause of death in the two dead FP cases. A patient with high malnutrition (ALB <30 g/L) and anemia (Hb = 86 g/L) died of severe heart failure after refusing treatment, and another with high malnutrition (ALB <30 g/L) and diabetes (more than 10 years) died from gastrointestinal bleeding after FP remission. In terms of the long-term outcomes between the two groups by the end of follow-up, there were still 7 cases in the FP group (46.7%) and 11 cases in the BP group (12.2%) receiving HD. Only one case still undergoing PD in the FP group (6.7%), while 59 cases (65.6%) in the BP group. A total of 2 cases received kidney transplantation, of which one (6.7%) was from the FP group and another (1.1 %) was from the BP group during the study period. Four cases died in the FP group (26.7%) and 17 cases in the BP group (18.9%).

**Table 3:** Comparison of patients with fungal versus bacterial peritonitis (1:6 ratio matching) [Mean ± SD, Median (P25, P75), *n* (%)].

**Variables**	**FP (*n* = 15)**	**BP (*n* = 90)**	***P* value**
Age	55.4 ± 15.4	55.0 ± 12.6	0.908
Age ≥ 65	5 (33.3)	20 (22.2)	0.543
Female gender	7 (46.7)	36 (40.0)	0.627
Body temperature (°C)	37.1 ± 0.7	37.0 ± 0.7	0.750
MAP (mmHg)	103.6 ± 24.5	99.9 ± 15.8	0.449
BMI (kg/m^2^)	21.6 (21.2, 25.0)	23.9 (21.6, 26.6)	0.270
PD duration (months)	28.6 (12.3, 58.8)	26.7 (6.9, 49.3)	0.516
Diabetes	6 (40.0)	29 (32.2)	0.554
CVD	3 (20.0)	24 (26.7)	0.820
Under immunosuppressive therapy	2 (13.3)	8 (8.9)	0.946
Previous bacterial peritonitis	4 (26.7)	37 (41.1)	0.288
Previous antibiotic use	15 (100.0)	25 (27.8)	** <0.001**
Potassium (mmol/L)	3.93 ± 0.87	3.82 ± 0.75	0.603
Calcium (mmol/L)	2.05 ± 0.30	2.13 ± 0.22	0.203
Phosphorus (mmol/L)	1.44 ± 0.49	1.43 ± 0.48	0.934
Hemoglobin (g/L)	104.5 ± 26.1	100.3 ± 22.1	0.510
Serum albumin (g/L)	30.9 ± 8.6	33.1 ± 4.9	0.152
Serum albumin <30 g/L	8 (53.3)	26 (28.9)	0.115
Glucose (mmol/L)	7.2 (4.6, 9.6)	8.1 (6.1, 9.3)	0.276
Serum creatine (μmol/L)	739 (659, 947)	907 (706, 1,047)	0.160
Blood urea nitrogen (mmol/L)	16.7 (11.0, 19.9)	16.4 (13.8, 21.9)	0.318
Blood uric acid (μmol/L)	385 (340, 442)	385 (341, 385)	0.324
eGFR (ml/min/1.73 m^2^)	5.2 (5.2, 6.6)	4.8 (3.8, 5.4)	0.100
iPTH (pg/ml)	255 (92, 346)	350 (180, 479)	0.179
hsCRP (mg/L)	95.1 (31.0, 127.7)	82.3 (34.7, 131.4)	0.830
Blood WCC (10^9^/L)	8.0 (6.4, 10.7)	7.9 (6.0, 10.9)	0.728
Peritoneal effluent WCC (10^6^/L)	1,020 (520, 2,100)	1,860 (750, 5,400)	0.093
Peritoneal effluent PMN (%)	80 (65, 88)	90 (83, 92)	** <0.01**
24 h urine output (ml)	350 (0, 700)	200 (86, 850)	0.985
Anuric	5 (33.3)	24 (26.7)	0.824
**Seasonal change**			
Spring	3 (20.0)	20 (22.2)	1.000
Summer	4 (26.7)	23 (25.6)	1.000
Autumn	5 (33.3)	36 (40.0)	0.624
Winter	3 (20.0)	11 (12.2)	0.682

*FP, fungal peritonitis; BP, bacterial peritonitis; MAP, mean arterial pressure; BMI, body mass index; PD, peritoneal dialysis; CVD, cardiovascular disease; eGFR, estimated glomerular filtration rate; iPTH, intact parathyroid hormone; hsCRP, high sensitivity C-reactive protein; EWCC, white cell count; PMN, polymorphonuclear; h, hour*.

*The boldface indicated that P <0.05 are considered statistically significant*.

**Table 4:** Clinical outcomes of patients with FP and BP [*n* (%)].

**Events**	**Short-term outcomes (<3 M)**		**Long-term outcomes (≥** **3 M)**	
	**FP (*n* = 15)**	**BP (*n* = 90)**	**FP (*n* = 15)**	**BP (*n* = 90)**
Death	2 (13.3)	2 (2.2)	4 (26.7)	17 (18.9)
HD transfer	10 (66.7)	8 (8.9)^**^	7 (46.7)	11 (12.2)^*^
PD resumption	3 (20.0)	81 (90.0)^**^	1 (6.7)	59 (65.6)^**^
Kidney transplant	0	0	1 (6.7)	1 (1.1)
Lost to follow-up	0	0	2 (13.3)	2 (2.2)

*FP, fungal peritonitis; BP, bacterial peritonitis; HD, hemodialysis; PD, peritoneal dialysis*.

* *The single asterisk indicated that P < 0.01 are considered statistically significant*.

** *The two asterisks indicated that P < 0.001 are considered statistically significant*.

Between the two groups, the FP group showed a higher rate of HD transfer and a lower rate of PD resumption (all *P* < 0.01) than the BP group, while no significant difference in the mortality was noted during the whole study period (*P* > 0.05), as shown in Table 4.

### Comparison of Risk Factors Between FP and BP in the 1:6 Ratio Cohort

As shown in Table 3, the results did not show differences in baseline data between the two groups except that the usage rate of antibiotics in 1 month before the onset of peritonitis was higher (100.0 vs. 27.8%, *P* < 0.001) and the median peritoneal effluent PMN was lower (80 vs. 90%, *P* < 0.01) in the FP group than those in the BP group.

The variables used for the univariate logistics regression model were age, sex, BMI, PD duration, diabetes, CVD history, under immunosuppressive therapy, previous BP, previous antibiotic use, serum potassium, hemoglobin, serum albumin, blood uric acid, eGFR, iPTH, hsCRP, peritoneal effluent PMN and season change (Supplementary Table S3). In the multivariable logistics regression analysis, we included age, sex, BMI, PD duration, diabetes, CVD history, previous BP, potassium, hemoglobin, serum albumin, eGFR, and peritoneal effluent PMN in the model, and these results indicated that longer PD duration [odds ratio (OR) 1.042, 95% confidence interval (CI) 1.012–1.073, *P* < 0.01], elevated serum potassium (OR 3.373, 95% CI 1.068–10.649, *P* < 0.05) and estimated glomerular filtration rate (eGFR) (OR 1.845, 95% CI 1.151–2.955, *P* < 0.05), and decreased serum albumin level (OR 0.820, 95% CI 0.695–0.968, *P* < 0.05) and peritoneal effluent PMN (OR 0.940, 95% CI 0.900–0.981, *P* < 0.01) were independent risk factors for FP onset, as shown in Table 5.

**Table 5:** Univariate and multivariate logistic regression analysis for risk factors of FP.

**Variable**	**Univariate**			**Multivariate**		
	**OR value**	**95% CI**	***P* value**	**OR value**	**95% CI**	***P* value**
Age (per 1 year)	1.003	0.961–1.046	0.907	0.993	0.925–1.065	0.838
Male gender	0.762	0.254–2.286	0.628	0.267	0.047–1.511	0.135
BMI (per kg/m^2^)	0.989	0.886–1.103	0.838	1.077	0.931–1.247	0.319
PD duration (per months)	1.005	0.988–1.022	0.550	1.042	1.012–1.073	**0.006**
Diabetes	1.402	0.456–4.313	0.555	2.315	0.498–10.754	0.284
CVD history	0.688	0.178–2.648	0.586	1.499	0.218–10.301	0.680
Previous BP	0.521	0.154–1.763	**0.294**	0.188	0.034–1.033	0.055
Potassium (per mmol/L)	1.209	0.596–2.449	0.599	3.373	1.068–10.649	**0.038**
Hemoglobin (per g/L)	1.008	0.984–1.033	0.507	1.035	0.995–1.078	0.089
Serum albumin (per g/L)	0.927	0.835–1.029	**0.154**	0.820	0.695–0.968	**0.019**
eGFR (per ml/min/1.73 m^2^)	1.292	1.002–1.667	**0.048**	1.845	1.151–2.955	**0.011**
Peritoneal effluent PMN (per %)	0.961	0.931–0.992	**0.013**	0.940	0.900–0.981	**0.005**

*The variables included in the logistics regression model were age, sex, BMI, PD duration, diabetes, CVD history, previous BP, serum potassium, hemoglobin, serum albumin, eGFR, and peritoneal effluent PMN*.

*OR, odds ratio; CI, confidence intervals; FP, fungal peritonitis; BP, bacterial peritonitis; BMI, body mass index; PD, peritoneal dialysis; CVD, cardiovascular disease; eGFR, estimated glomerular filtration rate; PMN, functionality of neutrophils; h, hour.*

*The boldface indicated that p < 0.05 are considered statistically significant*.

### Literature Review

We conducted a comprehensive literature review of the clinical studies on FP in patients on PD. Table 6 shows a representative study of FP in patients on PD. Overall peritonitis and FP incidence rates in our center appear to be improved or similar to previous studies (6–11, 19). In addition, this study is one of the few long-term case-control studies with relatively larger sample sizes which focused on analyzing the clinical characteristics of FP, comparing outcomes of FP vs. BP, and estimating risk factors for FP on PD patients in southern China.

**Table 6:** Previous and present studies focusing on fungal peritonitis in peritoneal dialysis.

**References**	**Shouci Hu (6)**	**Sara Auricchio (7)**	**Aydin Unal (8)**	**Ruchir Chavada (18)**	**Jun Ni (9)**	**Hong Qing Cui (11)**	**Hong Wang (10)**	**Present study**
Country	China	Italy	Turkey	Australia	China	China	China	China
Design	Single-center, retrospective	Single-center, retrospective	Single-center, retrospective	Two centers, retrospective	Single-center, retrospective	Single-center, retrospective	Single-center, retrospective	Single-center, retrospective
Study Duration (years)	5	34	15	9	10	8	6	10
PD patients (*n*)	730	None	None	2,075	None	635	None	739
Peritonitis episodes (*n*)	436	589	None	1,568	542	248	241	268
Peritonitis rate (episodes per patient-year)	0.266	0.444	None	0.8	None	None	None	0.127
FP episodes (*n*)	11	14	21	39	24	19	16	15
FP/PDAP (%)	2.5	2.4	None	2.5	4.4	7.7	6.6	5.6
PD duration (months) [mean ± SD, M (P25, P75)]	43 (22, 52)	45.6 ± 50.4	48 (9–95)	37.8 (15.5–57)	65.5 (27.75, 96.25)	10–96 (45.2 ± 25.7)	31.3 ± 37.6	28.6 (12.3, 58.8)
FP rate (episodes per patient-year)	0.0067	None	None	0.02	None	None	None	0.0071
Previous antibiotic treatment (*n*, %)	5 (45.5)	14 (100)	21 (100)	20 (51)	10 (41.7)	17 (89.5)	11 (68.8)	6 (40)
Previous bacterial peritonitis (*n*, %)	5 (45.5)	11 (78.6)	4 (19.0)	20 (51.0)	20 (83.3)	17 (89.5)	None	4 (26.7)
Major causative organisms	*C. albicans* (6/11)	*C. parapsilosis* (7/14)	*C. albicans* (14/21)	*C. albicans* (14/39)*, C. parapsilosis* (12/39)	*C. parapsilosis* (9/25)	*C. albicans* (7/19), *C. parapsilosis* (6/19)	*C. albicans* (4/16)	*C. albicans* (7/15)
Major antifungal treatments	fluconazole (9/11)	fluconazole (13/14)	amphotericin B (19/21)	fluconazole (33/39)	fluconazole (17/24)	fluconazole (16/19)	fluconazole (16/16)	fluconazole (14/15)
prophylaxis treatment against FP	No	No → Yes	Yes	No	No	No	Yes	No
Catheter removal (*n*, %)	8 (72.7)	14 (100)	21 (100)	31 (79)	22 (91.7)	19 (100)	16 (100)	13 (86.7)
Median duration until PD catheter removal (days)	5.5 (4.0, 11.0)	4 (1, 8)	1 (0, 10)	None	6 (2, 10)	2 (1, 3)	None	3 (0, 6)
Death (*n*, %)	4 (36.4)	2 (14.3)	2 (9.5)	6 (15)	6 (25.0)	0	1 (6.3)	2 (13.3)
HD transfer (*n*, %)	6 (54.5)	14 (100)	19 (90.5)	None	17 (70.8)	19 (100)	None	10 (66.7)
PD resumption (*n*, %)	1 (9)	14 (7.1)	0	None	1 (4.2)	0	None	3 (20)
Hospitalization (days) [mean ± SD, M (P25, P75)]	22 (17, 30.5)	27 ± 19	21-28	24	30 (27.5–45.0)	None	None	18 (10, 38)
Study group	FP vs. BP (11/55)	None	None	FP vs. BP (39/78)	FP vs. BP (24/96)	FP vs. BP vs. control (19/229/347)	FP vs. G+ vs. G– (16/126/45)	FP vs. BP (15/45)

*PD, peritoneal dialysis; FP, fungal peritonitis; BP, bacterial peritonitis; HD, hemodialysis; PDAP, peritoneal dialysis-associated peritonitis*.

## Discussion

In this 10-year single-center, retrospective, observational and case-control study, we analyzed 15 cases of FP diagnosed in our hospital. To better explore the risk factors of FP outbreak, we matched FP and BP patients in 1:6 ratio. Our results showed that the peritonitis and FP incidence rate in our hospital was similar or even lower than that in other centers, and the FP patients had significantly worse clinical outcomes than BP patients. We also found that FP group had a higher rate of previous antibiotic use than that in BP group, and longer PD duration, elevated potassium and eGFR level, and decreased serum albumin level and peritoneal effluent PMN were independent risk factors for FP.

FP is a relatively rare but severe complication in patients on PD. The current literature reported that FP incidence accounted for 2.4–7.7% of PDAP (Table 6), with an FP rate of 0.0067–0.02 episodes per patient-year (12, 20). Our review showed 15 cases (5.6%) of FP in a total of 268 episodes of peritonitis, slightly higher than reported in the literature. However, we recorded a very low PDAP rate (0.127 episodes per patient-year) compared to 0.266–0.444 episodes per patient-year reported by other centers (6, 7, 12). Our 10-year FP rate was also very low (0.0071 episodes per patient-year) and decreased from 0.0170 to 0.0051 episodes per patient-year between the first (2011–2015) and the second (2016–2020) 5-year periods.

As reported, the etiology of FPs is mainly *Candida*, accounting for about 70–90% of adult FPs (12, 14), among which *C. albicans* is the majority. Variations in fungal pathogen profiles can be related to factors such as the population involved, geographical area, previous antifungal exposure, and age of patients (21). All FP cases in our center were caused mainly by *Candida*, of which *C. albicans* accounted for the most of the cases, consistent with other reports. We also found that *C. parapsilosis* was the second most frequent pathogen in the samples. Recent studies reported changes in the prevalence of pathogenic yeast, indicating that non-albicans yeasts, represented by *C. parapsilosis*, have been on the rise (7, 9, 14). In regards to the treatment, our study observed varying susceptibility patterns. The intermediate range for itraconazole in two isolates of *C. albicans* and 5-flucytosine in one *C. albicans* were observed. The other 13 strains were all susceptible to fluconazole, voriconazole, 5-flucytosine, itraconazole, and amphotericin B.

The 2016 ISPD guidelines (5) recommend antifungal prophylaxis while PD patients receive antibiotic courses to prevent FP. A large number of studies (22, 23) have examined the use of oral nystatin or fluconazole as prophylaxis against FP, administered during antibiotic therapy, with contradictory results. In general, we do not use any antifungal prophylactic agent except in cases where FP is confirmed or highly suspected for our patients on PD. Our results showed that it did not increase FP incidence and poor prognosis without antifungal prophylactic agent, suggesting that non-prophylaxis of antifungal treatment is available under the premise of standard operation.

Appropriate antifungal administration and timely catheter removal lay the foundation for FP therapy (18, 24). Fluconazole is the most commonly used drug in the initial empiric treatment of FPs. In our center, 14 FP patients received initial treatment with fluconazole followed by an antifungal regimen for at least 14 days. As an exception, a patient with *Aspergillus fumigatus* complicated by bacterial infection received levofloxacin. Any complications associated with fluconazole use were reported, and all fungal strains were susceptible to fluconazole, but the side effects, such as QT interval prolongation or hepatotoxicity and the increasing rate of azole resistance (25), cannot be ignored.

Immediate catheter removal after identification of fungi was recommended according to the ISPD 2016 guidelines (5), as some studies (20, 26) indicate that catheter removal improves the outcome and reduces mortality. Because fungi can colonize the catheter creating a biofilm along its surface, all cases of FP, except two, underwent catheter removal without short-term complications. A patient who remained the catheter refused to be treated in our hospital and transferred to local hospital for treatment, finally he died of heart failure before catheter removal in 10 days. Another patient strongly refused to remove the catheter because the symptoms and signs of peritonitis were not obvious, and this patient temporarily stopped PD treatment until the peritoneal effluent microbiological test turned to be negative after anti-fungal therapy for 14 days and resumed PD again.

Removal of the catheter without antifungal therapy may increase the risk of peritoneal adhesion and thus deprive the patient of the opportunity to restart PD. The PD resumption rate in a North American center was 33%, while only 9.1% in a North China center. In our study, three FP cases (20%) were still on PD after an uneventful recovery, consistently to other studies, suggesting the importance of standardizing the treatment of FPs and reassessing the suitability of PD after FPs. In contrast, 10 cases (66.7%) switched to HD more than 3 months after catheter removal. From 2005, the presence of FP strongly recommended immediate catheter removal and HD transfer, as the guidelines showed (5, 18, 24). Some studies (27, 28) also suggested that pure medication could be used for treatment and catheter removal delayed until dialysate effluent is no longer cloudy for patients who are too old or too frail to support HD transfer. A particular case of a young patient without underlying diseases or obvious clinical symptoms and signs of peritonitis was infected with *C. albicans* in our study, and he was only given PD suspension and antifungal treatment, which eventually cured FP and restored PD. Hence, it is important to consider whether drug therapy is also a possible treatment for young patients with fewer underlying diseases, better physical fitness, and mild clinical symptoms when patients subjectively refuse to remove catheters or undergo HD. The worldwide mortality rate of FP is variable, ranging between 0 and 36.4% (Table 6). In our study, two cases (13.3%) died within 3 months after FP diagnosis. Some well-known risk factors related to mortality, including malnutrition, anemia, diabetes, and comorbidities, can lead to a poor prognosis of FP by exacerbating the patient's physical condition, so it was difficult to make sure whether if the occurrence of FP is directly associated with mortality or not.

The current studies (6, 13) revealed that previous use of antibiotics, previous BP, under immunosuppression, higher burden of comorbidities (such as diabetes, malnutrition), prolonged PD duration and loss of residual kidney function are common risk factors for FP. Due to the overgrowth of gastrointestinal fungi and the decline of peritoneal defenses, FP is more likely to develop secondary to antibiotics exposure (7). Based on the 2016 ISPD guidelines (5), PD patients with peritonitis must be treated with antibiotic treatment before the diagnosis of FP. Thus, all 15 cases of the FP group had received antibiotic therapy in the past 1 month in this study. The rate of antibiotic use history was much higher in FP group than that in the BP group (100.0 vs. 27.8%, *P* < 0.001). It suggests that we need to be alert to the possibility of FP once PD patients receive antibiotics, especially when antibiotic treatment is not effective. In this study, multivariate analysis showed that longer PD duration and decreased serum albumin level were the major risk factors for FP, consistent with current researches, indicating that patients with prolonged time on PD, malnutrition and hypoalbuminemia should be paid more attention to prevent FP.

Our study found that elevated serum potassium was an independent risk factor for FP. Further analysis indicated that there was no difference in the mean serum potassium level between the FP and BP groups (3.92 vs. 3.82 mmol/L, *P* > 0.05) in our 1:6 ratio cohort, and no case in the FP group had hyperkalemia (serum potassium > 5.50 mmol/L) while four patients experienced hyperkalemia in the BP group. Interestingly, most of previous studies (29–31) reported that serum potassium <3.5 mmol/L, particularly persistent hypokalemia, is associated with a higher risk of peritonitis caused by any organism. Given the inconsistent results of previous studies and our present study, we speculate that the reasons are as follows. First, the sample size of the FP group is limited, and it maybe affect the statistical efficiency. Second, it exists retrospective information bias using case-control study. Finally, the relationship of serum potassium level with peritonitis may differ depending on how hypokalemia/hyperkalemia is measured and over what time of PD duration. Therefore, the association between increased serum potassium or hyperkalemia and incidence of FP remains largely unknown and needs to be further explored. In addition, lower median peritoneal effluent PMN in the FP group were observed in FP patients when compared with BP (80 vs. 90%, P <0.01), and decreased peritoneal effluent PMN was independent risk factors for FP onset in our study, which maybe the inflammatory response of FP is not typical compared to that of BP. Notably, some studies believed that decreased residual renal function is a risk factor for the occurrence of FP, while data from our center found that elevated eGFR is one of the risk factors for FP. This may be related to the small sample size in the FP group in our center and the conclusion is worthy of further discussion. Furthermore, seasonal changes may influence the occurrence of peritonitis by changing the patients' health and the microenvironment in recent study (32), while no significant effects were noted between the occurrence of FP and the seasons in our study. It deserves further investigation.

Several limitations are acknowledged. First, this was a retrospective and observational study based on registry data, the type of peritonitis infection (FP vs. BP) was not randomly assigned. Although case-control method was used to select a 1: 6 matching cohort, which was always *post hoc*. There may be selection biases between the two groups that affected the effectiveness of outcome and risk factor comparisons. Second, whether our results are generalizable to other populations is uncertain because our patients were from a single center in Southern China, and the number of samples was relatively limited. Therefore, center-specific effects are inevitable. Despite these limitations, this is one of the few long-term case-control analyzing the characteristics of FP patients in mainland China. We reported a low incidence rate of overall peritonitis and FP. In addition, we conducted a comprehensive literature review of the published clinical studies on FP.

In conclusion, FP is a rare complication in patients on PD, leading to higher rates of catheter loss and HD transfer, and a lower rate of PD resumption than BP. Additional attention should be paid to those risk factors including hypoalbuminemia, increased serum potassium, long PD duration, and low peritoneal effluent PMN in PD patients. The best approach to improve clinical outcomes for FP is a prompt diagnosis, targeted antifungal therapy, and rapid catheter removal.

## Data Availability Statement

The original contributions presented in the study are included in the article/Supplementary Materials, further inquiries can be directed to the corresponding authors.

## Ethics Statement

The study has been approved by the Institutional Ethics Review Boards of Guangdong Provincial Hospital of Chinese Medicine, the Second Affiliated Hospital of Guangzhou University of Chinese Medicine, Guangzhou, China. The patients/participants provided their written informed consent to participate in this study.

## Author Contributions

RL and DZ contributed equally to this work and they performed the drafting of the work. JH, JO, XH, JW, and HL provided substantial contribution to the acquisition. YP, YX, and HH performed analysis and interpretation of data for the research. FL and XL conceived and designed the study. LZ contributed mainly to the analysis and interpretation of the supplementary data in the revised manuscript, and performed a critical review of the latest version of our manuscript. FL provided the final approval of the version to be published and agreed to be accountable for all aspects of the work.

## Funding

This work was financially supported by the State Key Laboratory of Dampness Syndrome of Chinese Medicine, the Second Affiliated Hospital of Guangzhou University of Chinese Medicine (Grant No. SZ2021ZZ43).

## Conflict of Interest

The authors declare that the research was conducted in the absence of any commercial or financial relationships that could be construed as a potential conflict of interest.

## Publisher's Note

All claims expressed in this article are solely those of the authors and do not necessarily represent those of their affiliated organizations, or those of the publisher, the editors and the reviewers. Any product that may be evaluated in this article, or claim that may be made by its manufacturer, is not guaranteed or endorsed by the publisher.

## Supplementary Material

Supplementary Table S1 Patient characteristics, biochemistry indices and clinical outcomes among the 15 cases of FP.

Supplementary Table S2 Patient characteristics, biochemistry indices and clinical outcomes among the 105 PD cases, including 15 cases of FP and 90 cases of BP.

Supplementary Table S3 Logistic univariate regression analysis for risk factors of fungal peritonitis.
