# Supplementary material for: Characteristics Analysis, Clinical Outcome and Risk Factors for Fungal Peritonitis in Peritoneal Dialysis Patients: A 10-Year Case-Control Study
Source: Front Med (Lausanne). 2021 Dec 1;8:774946. doi: 10.3389/fmed.2021.774946 (PMC8671457; doi:10.3389/fmed.2021.774946)
Supplement: Supplementary Table S3 — Logistic univariate regression analysis for risk factors of fungal peritonitis. [file Table_3.docx]

**SUPPLEMENTARY TABLE S3 |** Logistic univariate regression analysis for risk factors of fungal peritonitis

| **Variables** | ***B* value** | **OR value** | **95% CI** | ***P* value** |
| --- | --- | --- | --- | --- |
| Age | 0.003 | 1.003 | 0.961 - 1.046 | 0.907 |
| Sex | -0.272 | 0.762 | 0.254 - 2.286 | 0.628 |
| BMI (kg/m2) | -0.011 | 0.989 | 0.886 - 1.103 | 0.838 |
| PD duration (months) | 0.005 | 1.005 | 0.988 - 1.022 | 0.550 |
| Diabetes | 0.338 | 1.402 | 0.456 - 4.313 | 0.555 |
| CVD history | -0.375 | 0.688 | 0.178 - 2.648 | 0.586 |
| Under immunosuppressive therapy | 0.455 | 1.577 | 0.301 - 8.263 | 0.590 |
| Previous BP | -0.652 | 0.521 | 0.154 - 1.763 | 0.294 |
| Previous antibiotic use | 20.692 | >999.999 | 0.000 - >999.999 | 0.997 |
| Potassium (mmol/L) | 0.190 | 1.209 | 0.596 - 2.449 | 0.599 |
| Hemoglobin (g/L) | 0.008 | 1.008 | 0.984 - 1.033 | 0.507 |
| Serum albumin (g/L) | -0.076 | 0.927 | 0.835 - 1.029 | 0.154 |
| Blood uric acid (μmol/L) | 0.004 | 1.004 | 0.996 - 1.011 | 0.339 |
| eGFR (ml/min/1.73m^2^) | 0.257 | 1.292 | 1.002 - 1.667 | **0.048** |
| iPTH (pg/mL) | -0.001 | 0.999 | 0.997 - 1.002 | 0.522 |
| hsCRP (mg/L) | 0.001 | 1.001 | 0.996 - 1.006 | 0.789 |
| Peritoneal effluent PMN (%) | -0.040 | 0.961 | 0.931 - 0.992 | **0.013** |
| **Seasonal change** |  |  |  |  |
| Winter | Reference | Reference | Reference | Reference |
| Spring | -0.598 | 0.550 | 0.094 - 3.201 | 0.506 |
| Summer | -0.450 | 0.638 | 0.121 - 3.355 | 0.595 |
| Autumn | -0.675 | 0.509 | 0.105 - 2.479 | 0.403 |

*The variables included in the univariate logistics regression model were age, sex, BMI, PD duration, diabetes, CVD history, under immunosuppressive therapy, previous BP, previous antibiotic use, serum potassium, hemoglobin, serum albumin, blood uric acide, GFR, iPTH, hsCRP, peritoneal effluent PMN and season change. Because the lowest incidence of peritonitis occurred during winter, the winter group was treated as the reference group.*

*OR: odds ratio; CI: confidence intervals; FP: fungal peritonitis; BP: bacterial peritonitis; BMI: body mass index; PD: peritoneal dialysis; CVD: cardiovascular disease; eGFR: estimated glomerular filtration rate; PMN: functionality of neutrophils; h: hour.*

*The boldface indicated that p values less than 0.05 are considered statistically significant.*
